# Frequent loss-of-function mutations in the AMPK-α2 catalytic subunit suggest a tumour suppressor role in human skin cancers

**DOI:** 10.1042/BCJ20230380

**Published:** 2023-12-01

**Authors:** Fiona A. Ross, Simon A. Hawley, Fiona M. Russell, Nicola Goodman, D. Grahame Hardie

**Affiliations:** Division of Cell Signalling & Immunology, School of Life Sciences, University of Dundee, Scotland, U.K.

**Keywords:** AMPK, melanoma, point mutations, skin cancer, tumour suppressors

## Abstract

The AMP-activated protein kinase (AMPK) is a sensor of cellular energy status activated by increases in AMP or ADP relative to ATP. Once activated, it phosphorylates targets that promote ATP-generating catabolic pathways or inhibit ATP-consuming anabolic pathways, helping to restore cellular energy balance. Analysis of human cancer genome studies reveals that the *PRKAA2* gene (encoding the α2 isoform of the catalytic subunit) is often subject to mis-sense mutations in cancer, particularly in melanoma and non-melanoma skin cancers, where up to 70 mis-sense mutations have been documented, often accompanied by loss of the tumour suppressor NF1. Recently it has been reported that knockout of *PRKAA2* in NF1-deficient melanoma cells promoted anchorage-independent growth *in vitro*, as well as growth as xenografts in immunodeficient mice *in vivo*, suggesting that AMPK-α2 can act as a tumour suppressor in that context. However, very few of the mis-sense mutations in *PRKAA2* that occur in human skin cancer and melanoma have been tested to see whether they cause loss-of-function. We have addressed this by making most of the reported mutations and testing their activity when expressed in AMPK knockout cells. Of 55 different mis-sense mutations (representing 75 cases), 9 (12%) appeared to cause a total loss of activity, 18 (24%) a partial loss, 11 (15%) an increase in phenformin-stimulated kinase activity, while just 37 (49%) had no clear effect on kinase activity. This supports the idea that AMPK-α2 acts as a tumour suppressor in the context of human skin cancer.

## Introduction

The AMP-activated protein kinase (AMPK) pathway acts as a sensor of cellular energy and nutrient status, and a central regulator of cell growth in eukaryotes [1]. AMPK acts in direct opposition to the mechanistic target-of-rapamycin complex-1 (mTORC1) pathway, being activated by lack of energy or nutrients and switching off cell growth and proliferation, while mTORC1 is activated by the availability of energy and nutrients and promotes cell growth [2]. Genes encoding subunits of the AMPK and mTORC1 complexes are found in the genomes of essentially all eukaryotes, suggesting that these opposing signalling pathways arose very early during eukaryotic evolution [2–4].

AMPK exists universally as heterotrimeric complexes comprising catalytic α subunits and regulatory β and γ subunits. In vertebrates, there are multiple isoforms of each subunit (α1, α2; β1, β2; γ1, γ2, γ3) encoded by distinct genes, which can give rise to up to 12 distinct heterotrimeric combinations [5]. The classical (or canonical) stimulus for AMPK activation is a falling cellular energy status, which is signalled by increases in cellular AMP : ATP ratios. Replacement of ATP by AMP at the CBS3 site (one of three adenine nucleotide binding sites on the γ subunit [6,7]; site numbering as in [8]), triggers conformational changes that promote AMPK activation by: (i) direct allosteric activation; (ii) promotion of phosphorylation at Thr172 in the activation loop of the kinase domain of the α subunit by the upstream kinase LKB1; and (iii) inhibition of Thr172 dephosphorylation by protein phosphatases [1,2]. Binding of ADP to the γ subunit can have similar effects, at least with some isoform combinations [9], although it does not appear to cause allosteric activation [10,11]. In response to other cellular stresses, AMPK can be activated by non-canonical mechanisms that trigger phosphorylation at Thr172 due to: (i) activation of the calmodulin-dependent kinase CaMKK2 in response to increases in cytosolic Ca^2+^, triggered by some hormones and cytokines [12–14]; (ii) activation of CaMKK2 in response to increases in nuclear Ca^2+^ triggered by DNA damage [15,16]; or (iii) co-localisation with LKB1 at the lysosome in response to glucose starvation, which (at least in some cell types) can occur in the absence of any changes in adenine nucleotides [17]. AMPK can also be allosterically activated by long chain acyl-CoA esters, which bind at the so-called Allosteric Drug and Metabolite (ADaM) site located between the N-lobe of the kinase domain of the α subunit and the Carbohydrate-Binding Module of the β subunit [18], a site that also binds several pharmacological activators [19].

Once activated, AMPK phosphorylates up to 100 downstream targets at serine or threonine residues surrounded by well-defined recognition motifs [1]. This has the overall effect of switching on alternate catabolic pathways that generate ATP, while switching off biosynthetic pathways as well as halting progress through the cell cycle; AMPK therefore inhibits cell growth and proliferation, processes that would otherwise consume large quantities of ATP. AMPK activation suppresses cell growth in part by inhibiting mTORC1 via phosphorylation of the upstream regulator TSC2 [20] and the mTORC1 subunit Raptor [21]. Interestingly, mTORC1 has recently been reported to reciprocally inhibit AMPK via direct phosphorylation of the α1 or α2 subunit, in both fission yeast and humans [22].

The finding that LKB1 was a key upstream kinase phosphorylating Thr172 [23–25] introduced a previously unanticipated link between AMPK and cancer [26]. LKB1 was originally identified genetically as the tumour suppressor mutated in Peutz-Jeghers syndrome, an inherited susceptibility to cancer in humans [27], but it is also frequently mutated in sporadic (non-inherited) cancers, especially lung adenocarcinomas [28–31]. Given that AMPK inhibits cell growth and proliferation, it seemed likely that it mediated at least some of the tumour suppressor effects of LKB1. Indeed, global knockout in mice of the *Prkaa1* gene (encoding AMPK-α1, the only catalytic subunit expressed in lymphocytes) accelerated development of lymphomas induced by B-cell specific over-expression of the *Myc* oncogene in mice [32]. Moreover, T cell-specific knockout of *Prkaa1* in mice resulted in earlier onset and more rapid development of T cell acute lymphoblastic leukaemia/lymphoma (T-ALL) that had been initiated by T cell-specific knockout of *Pten* [33]. In the latter study, disease onset was also delayed by *in vivo* treatment with the AMPK-activating drug phenformin, an effect that required the presence of AMPK-α1 in the developing T cells. These studies show that AMPK can act as a tumour suppressor in the context of B- and T-cell lymphomas in mice. However, other studies showed that if the *Prkaa1* gene was knocked out in mice *after* T-ALL had arisen the disease was ameliorated, showing that in that context AMPK-α1 could act as a tumour promoter [34]. In mouse models of non-small cell lung cancer induced by mutations in K-Ras and/or p53, simultaneously knocking out both AMPK-α1 and -α2 was reported to either exacerbate [35–37] or ameliorate [38] the disease, although more prominent effects, similar to those obtained by knocking out LKB1, were obtained by knocking out the AMPK-related kinases SIK1-3 [37,39]. Overall, these apparently conflicting results can be rationalised by proposing that while AMPK acts as a tumour suppressor that can protect against the development of cancer, once a cancer has arisen it switches from being a tumour suppressor to a tumour promoter, perhaps because it protects tumour cells against the energetic, nutritional or oxidative stresses that they experience due to their rapid growth [30,40].

Thus, AMPK can either suppress or promote cancer in mice, depending on the timing of its loss and the cellular context. Analysis of genetic changes in AMPK genes in human cancers using the cBioPortal database [41,42] revealed similar dichotomies. While the *PRKAA2* gene (encoding AMPK-α2) is subject to relatively frequent mis-sense mutations in human cancers, the *PRKAA1* gene is rarely mutated but is instead often amplified, especially in lung adenocarcinomas [30,31]. For reasons that remain unclear, mutations in the *PRKAA2* gene (encoding AMPK-α2) are particularly frequent in melanoma and non-melanoma skin cancer — the top six human cancer genome studies that detected the most frequent *PRKAA2* mutations, in which the frequency varied from 8% to 23% of cases, all involved skin cancer [30]. This suggests that AMPK-α2 might be acting as a tumour suppressor in these cancers, so that tumour cells with mutations in *PRKAA2* were being selected for. If this is correct, it would be expected that a large proportion of these mutations would result in loss of, or at least reduced, kinase activity, with a smaller proportion being neutral mutations that did not affect the kinase activity.

While this study was in progress, Yuan et al. [43] pointed out that *PRKAA2* mutations were relatively frequent in human melanoma (9% of all cases), often in combination with mutations in the tumour suppressor NF1. They also showed that CRISPR–Cas9 mediated knockout of *PRKAA2* in NF1-mutant melanoma cells promoted anchorage-independent growth in soft agar, while ectopic expression of *PRKAA2* in NF1- and AMPK-α2-deficient human melanoma cells greatly reduced anchorage-independent growth. Finally, knockout of *PRKAA2* in NF1-mutant melanoma cells increased their growth as xenografts in immunodeficient mice, and increased the presence of metastases in brain when they were administered by intracardiac injection. These results strengthen the idea that AMPK-α2 is acting as a tumour suppressor in the context of human melanoma, with loss-of-function mutations being selected for. However, this study [43] did not address what proportion of AMPK-α2 mutations occurring in melanoma and other types of skin cancer caused loss of kinase activity, a deficiency we have now addressed.

## Results

### Bioinformatic analysis of *PRKAA2* mutations in skin cancer

**Figure 1.**
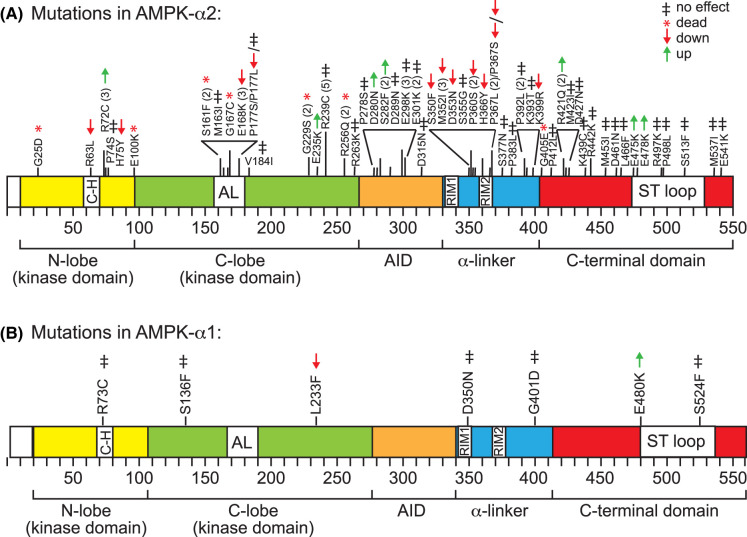
Mis-sense mutations reported to occur in all studies of skin cancer and melanoma listed in the cBioPortal database in mid-2019 for (A) AMPK-α2 and (B) AMPK-α1. The locations of the mutations are superimposed on domain diagrams that are drawn approximately to scale. Numbers in parentheses indicate the number of times (if >1) the mutation occurred during searching of the cBioPortal database. Abbreviations: C-H, C-helix in the N-lobe of the kinase domain; AL, activation loop of the kinase domain; AID, auto-inhibitory domain; RIM1/2, Regulatory Interaction Motifs 1 and 2, ST loop, serine/threonine-rich loop of the C-terminal domain. The effect of the mutation on phenformin-stimulated kinase activity in HEK-293 cells (see Table 2) are also summarised here for convenience, using the symbols shown at top right.

To provide an unbiassed selection to study, we decided to analyse all mis-sense mutations in the *PRKAA1* (encoding AMPK-α1) and *PRKAA2* (encoding AMPK-α2) genes that had been reported to occur in melanoma and non-melanoma skin cancer in the cBioPortal database at the time of commencement of our study in 2019. Figure 1A,B show the locations of mis-sense mutations identified at that time, drawn on domain diagrams of AMPK-α2 and AMPK-α1. In addition to these mis-sense mutations, in the case of *PRKAA2* there were a total of ten nonsense or frameshift mutations, or mutations affecting RNA splicing (Table 1). One splice site mutation was observed twice and another five times, making fifteen truncating mutations altogether. Of these, nine would cause truncations within the kinase domain that would clearly be non-functional, while the remainder would produce truncated α subunits lacking the C-terminal domain, which is required for the formation of the AMPK heterotrimer [44].

**Table 1:** Summary of 15 mutations (other than mis-sense mutations) in AMPK-α2 in skin cancer and melanoma, listed in the cBioPortal database in mid-2019

No.	Mutation	Type	Number of cases
1	G98*	Nonsense	1
2	R138*	Nonsense	1
3	F158=/X158_splice	Splice site	5
4	G159E/X159_splice	Splice site	2
5	K379*	Nonsense	1
6	A400Pfs*24	Frameshift	1
7	R407*	Nonsense	1
8	Q409*	Nonsense	1
9	X432_splice	Splice site	1
10	X474_splice	Splice site	1

**Table 2:** Summary of 55 mis-sense mutations in AMPK-α2 in skin cancer and melanoma (listed in the cBioPortal database in 2019) that were generated for this study

No.	Mutation	Cancer type	Cases	Domain	Location	Effect	PKA	AMPK-α1
1	G25D	M	1	KD	Glycine-rich loop	↓↓↓	Conserved	Conserved
2	R63L	CSCC	1	KD	αC	↓	N	Conserved
3	R72C	CM, BCC	3	KD	αC–β4	↑	N	Conserved
4	P74S	M	1	KD	αC–β4	-	Conserved	Conserved
5	H75Y	MM	1	KD	αC–β4	↓	F	Conserved
6	E100K	MM	1	KD	αD	↓↓↓	Conserved	Conserved
7	S161F	CM	2	KD	Activation loop	↓↓↓	A	Conserved
8	M163I	CM	1	KD	Activation loop	-	R	Conserved
9	G167C	CM	1	KD	Activation loop	↓↓↓	R	Conserved
10	E168K	CM	3	KD	Activation loop	↓	T	Conserved
11	P177L	CM	1	KD	Activation loop	-	Conserved	Conserved
12	P177S	CM	1	KD	Activation loop	↓	Conserved	Conserved
13	V184I	CM	1	KD		-	I	Conserved
14	G229S	CM/CSCC	2	KD	αG–αH	↓↓↓	Conserved	Conserved
15	E235K	CM	1	KD	αG–αH	↑	S	Q
16	R239C	CM/CSCC/BCC	5	KD	αH	-	S	P
17	R256Q	CM	1	KD	αH–αI	↓↓↓	Conserved	Conserved
18	R263K	CM	1	KD	αI	-	K	Conserved
19	P278S	CM	1	AID		-		Conserved
20	D280N	CM	1	AID		↑		Conserved
21	S282F	CM	2	AID		↑		Conserved
22	D289N	CM	1	AID		-		Conserved
23	E298K	CM/BCC	3	AID	α1	-		Conserved
24	E301K	CM	2	AID	α1–α2	-		Conserved
25	D315N	CM	1	AID	α2–α3	-		N
26	S350F	CM	1	α-linker		↓		Conserved
27	M352I	CM	3	α-linker		↓		L
28	D353N	DM	1	α-linker		↓		Conserved
29	S355G	CSCC	1	α-linker		-		H
30	P360S	DM	2	α-linker		↓		T
31	H366Y	CM	1	α-linker	RIM2	↓		Conserved
32	P367L	CM	2	α-linker	RIM2	↓		Conserved
33	P367S	CM	1	α-linker	RIM2	↓		Conserved
34	S377N	CM	1	α-linker		-		T
35	P383L	MM	1	α-linker		-		T
36	P392L	CM/CSCC	2	α-linker		-		S
37	K393T	MM	1	α-linker		-		Conserved
38	K399R	CSCC	1	α-linker		↓		Conserved
39	G405E	CM	1	α-linker		↓↓↓		Conserved
40	P412L	CM	1	CTD	α-helix-A	-		Conserved
41	R421Q	CM/MM	2	CTD	α-helix-A	↑		Conserved
42	M423I	CM	1	CTD	α-helix-A	-		I
43	D427N	CM	1	CTD	α-helix-A	-		Conserved
44	R439C	CM	1	CTD	β3 strand	-		Conserved
45	R442K	CM	1	CTD	β3 strand	-		Conserved
46	M453I	CM	1	CTD	β4-strand	-		Conserved
47	D461N	CM	1	CTD	β4/β5 loop	-		Conserved
48	L466F	CM	1	CTD	β5-strand	-		Conserved
49	E475K	CM/CSCC	2	ST loop		↑		Conserved
50	E478K	CSCC	1	ST loop		↑		Conserved
51	R497K	CM	1	ST loop		-		S
52	P498L	CM	1	ST loop		-		C
53	S513F	CM	1	ST loop		-		E
54	M537I	CM	1	CTD	Final α-helix	-		I
55	E541K	DM	1	CTD	Final α-helix	-		Conserved

Key to column ‘Cancer type’: M, melanoma; CM, cutaneous melanoma; MM, metastatic melanoma; DM, desmoplastic melanoma; CSCC, cutaneous squamous cell carcinoma; BCC, basal cell carcinoma. Key to column ‘Domain': KD, kinase domain; AID, auto-inhibitory domain; α-linker, linker between AID and CTD; CTD, C-terminal domain. Key to column ‘Effect’: ‘↓↓↓’, activity negligible; ↓, activity down-regulated; ‘-’, activity not affected; ‘↑’, activity up-regulated. Key to columns ‘Residue in PKA/Residue in α1’: ‘conserved' means the residue in that position is identical, otherwise the amino acid that replaces it in the Cα subunit of bovine PKA (kinase domain only) or AMPK-α1 is listed.

Taking into account situations where the same mutation had been reported in more than one case of melanoma or non-melanoma skin cancer, the overall frequency of mis-sense mutations in the AMPK-α2 sequence was 0.14 mutations/residue. However, there were ‘hot spots' or clusters where the frequency of mutation was higher (Figure 1A), such as the activation loop (AL) of the kinase domain that contains the critical phosphorylation site Thr172 (0.50 mutations/residue), the central part of the auto-inhibitory domain (AID) that causes inhibition of AMPK in the absence of AMP [45] (0.31 mutations/residue), and Regulatory Interaction Motif-2 (RIM2), which contacts AMP when it is bound in the critical site CBS3 on the γ subunit [46], as well as residues just upstream of it (0.43 mutations/residue).

In contrast, compared with 90 mis-sense plus truncating mutations in *PRKAA2* (0.16 per residue) there were just seven mis-sense mutations (0.013 per residue) in the *PRKAA1* gene (encoding the AMPK-α1 isoform) reported in melanoma or non-melanoma skin cancer, and there were no mutations at all affecting the ‘hot-spots' identified in the AMPK-α2 gene, i.e. the activation loop (AL), AID or RIM2 region of the α-linker (Figure 1B).

### A large proportion of the mutations in AMPK-α2 cause loss-of-function

To assess the functional effects of the mis-sense mutations in the *PRKAA2* gene that are associated with melanoma or non-melanoma skin cancer, we used HEK-293 cells with a double knockout of AMPK-α2 and -α1 created using the CRISPR/Cas9 method and expressed in them either wild type or mutant FLAG-tagged AMPK-α2 by transient transfection. Of 70 mis-sense mutations in *PRKAA2* in total, 15 did not express sufficiently well to allow analysis (some of these are also likely to cause loss-of-function *in vivo*, but this could not be tested using our methodology). This left 55 mutations that were further analysed — due to technical limitations, it was not feasible to analyse that many simultaneously, so we tested them in batches of up to 12, using cells expressing wild type AMPK-α2 as controls in the same experiment. Cells were treated with or without 10 mM phenformin for 1 h to activate AMPK by the canonical mechanism [47], and lysates were prepared. We first carried out Western blotting using anti-FLAG antibodies to determine the level of AMPK-α expression, and then immunoprecipitated a volume of lysate that would yield an equal amount of AMPK-α subunit in each precipitate.

**Figure 2.**
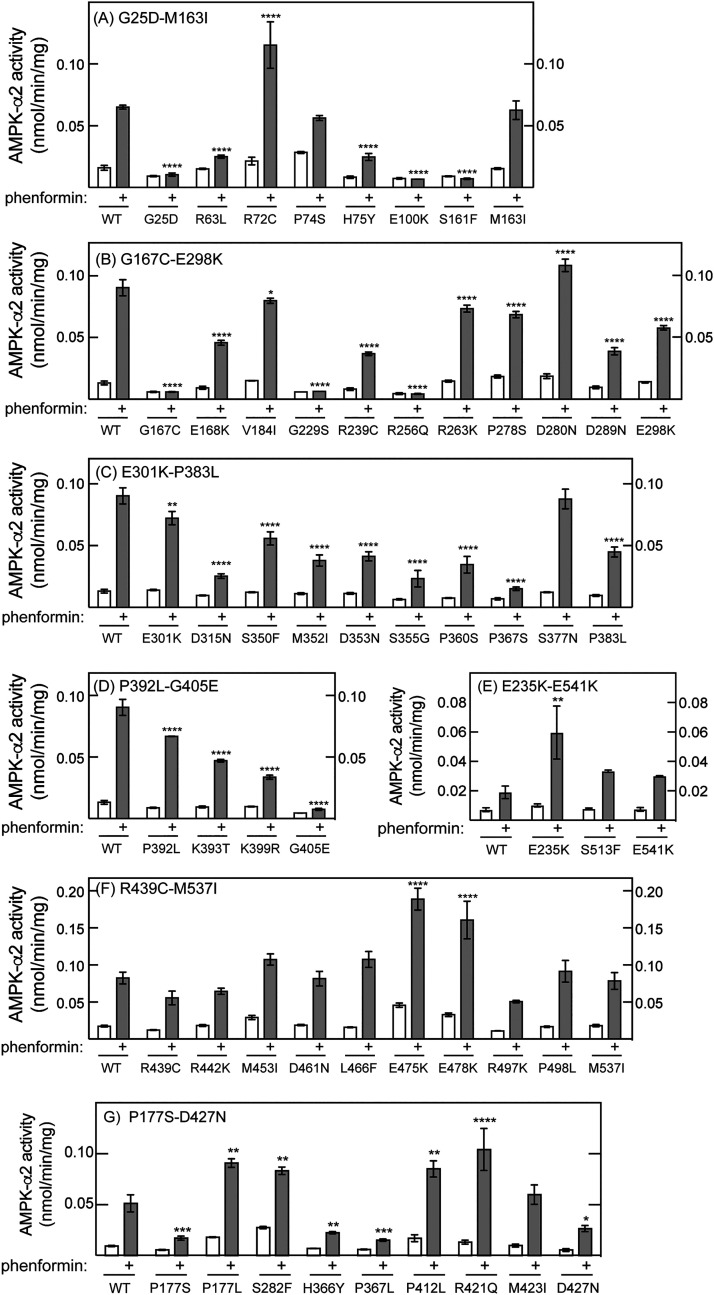
Effect of mis-sense mutations in AMPK-α2 on phenformin-stimulated AMPK activity following transfection into AMPK-α1/-α2 double knockout cells. Results are mean ± SEM for triplicate dishes for each mutant, with asterisks (* *P* < 0.05, ** *P* < 0.01, *** *P* < 0.001, **** *P* < 0.0001) denoting the significance of differences between phenformin-treated mutant and wild type (WT) cells. Within each panel [(**A**) to (**G**)], assays were conducted with the WT and all mutants shown at the same time. The mutants shown in (**E**) and (**G**) took longer to generate so their analyses were not performed in strict order of residue number,.

Figure 2A–G shows results for AMPK-α2 mutants from triplicate dishes of control and phenformin-treated cells. All analyses were repeated at least once to confirm that we had not made any errors in labelling of mutants, and that any changes in function observed were robust and reproducible. To be counted as down- or up-regulated (reduced or increased activation by phenformin) in Table 2, significant differences between the activity after phenformin treatment of the mutant compared with the wild type had to be obtained in at least two repeats of the experiment. This means that we might have missed differences with some mutants where the effects were quite subtle. The overall results are summarised in Table 2 and also Figure 1A.

The figures that follow take into account mutations that are identical but occurred independently in more than one case of skin cancer or melanoma — for example, S161F, which exhibited no significant activation by phenformin, was reported in two cases and was therefore counted as two mutations. Of the 75 cases of mis-sense mutations (comprising 55 different mutations) that expressed sufficiently well and were tested, 9 (12%) showed minimal activity and no activation by phenformin, 18 (24%) showed reduced activation by phenformin, and 37 (49%) showed no effect and were activated to the same extent by phenformin as the wild type. Interestingly, 11 (15%) were associated with a larger activation by phenformin than the wild type — one of these (R72C) occurred independently three times.

For the *PRKAA2* gene in skin cancer and melanoma, the database also contained five nonsense mutations, eight frameshift mutations and two mutations expected to cause splicing errors (Table 1), making 15 mutations that would all be expected to cause a total loss in AMPK-α2 function. Taking these into account also, 27% of mutations would be expected to cause total loss-of function, 20% partial loss-of function and 12% gain-of-function, with only 41% being apparently neutral mutations.

**Figure 3.**
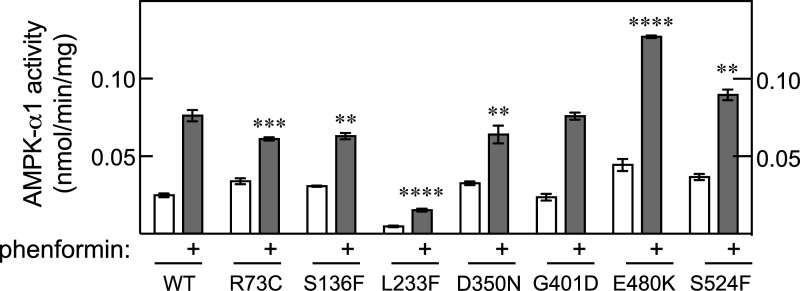
Effect of mutations in AMPK-α1 on phenformin-stimulated AMPK activity following transfection into AMPK-α1/-α2 double knockout cells. As Figure 2 but analysing all seven mis-sense mutations in AMPK-α1 reported in melanoma and non-melanoma skin cancer.

Figure 3C shows that none of the seven mutations in AMPK-α1 caused total loss-of-function. One (L233F) appeared to cause a reduced activity while another (E480K) caused increased activity compared with the wild type. Interestingly, the E480K mutation aligns with the E475K mutation in AMPK-α2, which was also associated with increased activation of α2-containing complexes by phenformin.

### Some mutations associated with loss of kinase activity in HEK-293 cells caused residual ACC1 phosphorylation in Hep-G2 cells treated with phenformin

**Figure 4.**
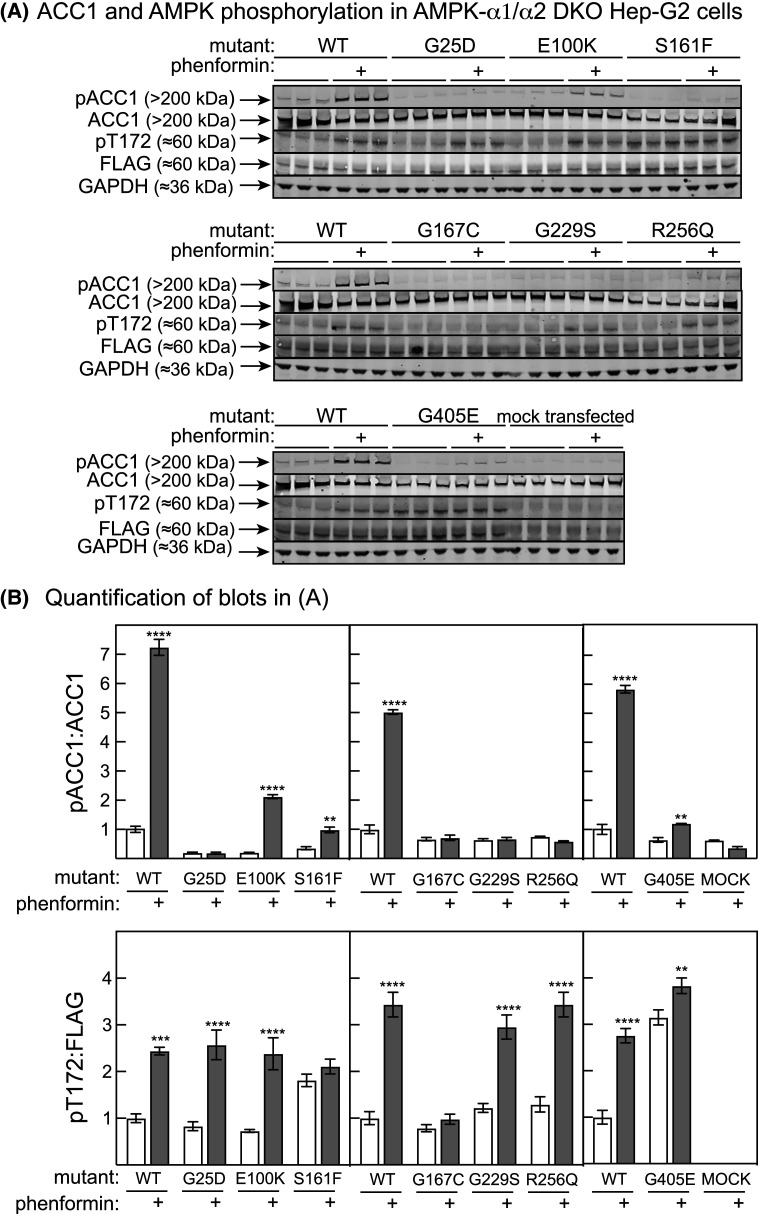
Phosphorylation of Ser80 on ACC and Thr172 in AMPK-α1/-α2 double knockout Hep-G2 cells transfected with potentially inactive FLAG-tagged mutants. (**A**) Western blots of cells transfected with the indicated mutants. Cells were transfected for 48 h and then treated with or without phenformin for 1 h prior to analysis. The experiments in each of the three panels was performed with triplicate dishes of cells at the same time. The molecular masses of the major bands detected, based on migration relative to SeeBlue™ Plus2 Pre-stained Protein Standards (ThermoFisher Scientific) are indicated, but these are approximate only. (**B**) Quantification of the results from (**A**): band intensities were estimated using a LI-COR Odyssey, and ratios of intensities for pACC:total ACC (top panel) and pT172:FLAG (bottom panel) are shown. Error bars are mean ± SEM (*n* = 3). Asterisks show cases where the ratios were significantly different in the presence and absence of phenformin.

We had originally planned to look at the phosphorylation of downstream targets such as acetyl-CoA carboxylase-1/-α (ACC1) in the double AMPK-α knockout HEK-293 cells used for Figures 2 and 3. However, we found that phenformin treatment caused some ACC1 phosphorylation even in mock-transfected cells, despite the fact that we could not detect significant AMPK activity in cell lysates following immunoprecipitation using a mixture of anti-α1 and -α2 antibodies (not shown). The explanation for this remains unclear, although we suspect it might be due to mutations occurring in the knockout cells during passaging that had restored some AMPK activity, sufficient to cause phenformin stimulation of ACC phosphorylation although not detectable in immunoprecipitate kinase assays. This technical problem did not affect the results in Figures 2 and 3 because we had analysed transfected AMPK in anti-FLAG immunoprecipitates (which would not detect any endogenous AMPK), but it did preclude analysis of phosphorylation of downstream targets such as ACC1 in these cells. We therefore analysed ACC1 phosphorylation in AMPK-α1/-α2 double knockout Hep-G2 cells that we had generated more recently, in which ACC1 phosphorylation was completely absent from mock-transfected cells (Figure 4). Using this approach, we analysed each of the seven individual mutations that had minimal basal activity and exhibited minimal activation by phenformin in Figure 2. The results (Figure 4) confirmed that activation of the wild type by phenformin was associated with increased phosphorylation of ACC1 at the AMPK site (Ser80) as expected. ACC1 phosphorylation was undetectable with the G25D, G167C, G229S and R256Q mutants with or without phenformin treatment, confirming that these four mutations cause a total loss of AMPK activity in intact cells. In the presence of phenformin, there was limited but detectable ACC1 phosphorylation with the E100K, S161F and G405E mutants, suggesting that these have a low kinase activity that was undetectable in our HEK-293 cell assays.

Of the seven mutations tested in Figure 4, four (G25D, E100K, G229S and R256Q) appeared to be phosphorylated normally at Thr172 on AMPK-α2 in response to phenformin treatment. However, G167C was not phosphorylated at Thr172 in response to phenformin, indicating that this replacement (affecting a residue just five residues upstream of Thr172) affects recognition by the upstream kinase LKB1. Interestingly, the other two mutants (S161F and G405E) appeared to be phosphorylated at Thr172 quite substantially even under basal conditions in the absence of phenformin.

### Phosphorylation and activation of bacterially expressed mutants by LKB1 and allosteric activation by MK-8722

**Figure 5.**
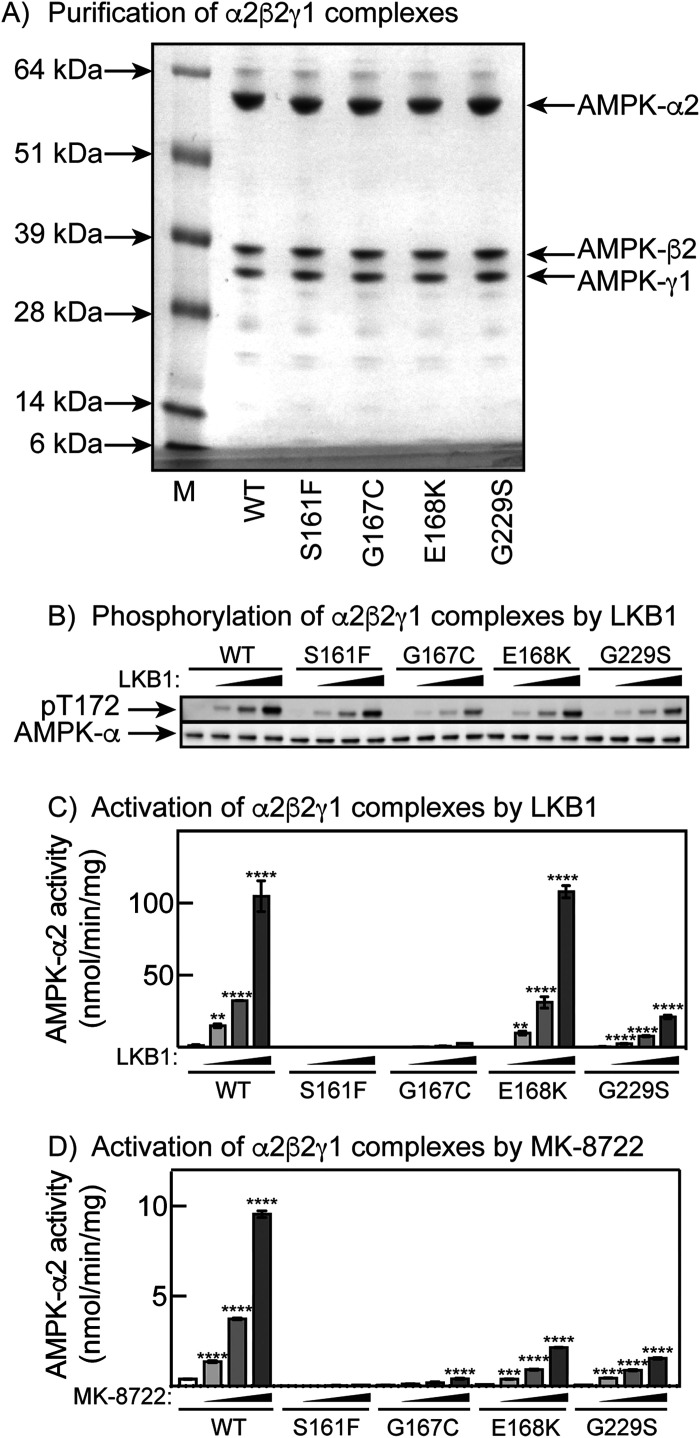
Phosphorylation and activation of activation loop mutants, and G229S, after their expression in and purification from bacteria. (**A**) Coomassie Blue-stained gel of WT and four mutants after purification on Ni^2+^-agarose. (**B**) Phosphorylation of Thr172 assessed using phosphospecific and phosphorylation-independent antibodies using increasing concentrations of the LKB1 complex as described in the Methods section. (**C**) As (**B**), but analysing AMPK activity; results are mean ± SEM (*n* = 2). (**D**) Allosteric activation of naïve, unphosphorylated α2β2γ1 complex by MK-8722, added (left to right) at 30 nM, 300 nM or 3 µM; results are mean ± SEM (*n* = 3).

As shown in Figure 1A, several mis-sense mutations in AMPK-α2 cluster in the activation loop containing Thr172, and we wished to confirm whether these were phosphorylated and activated by the upstream kinase using an alternative approach. We expressed the human α2β2γ1 complex and three activation loop mutants (S161F, G167C and E168K) in *Escherichia coli,* and purified the complexes using the (His)_6_ tag at the N-terminus of the α subunit. We also did the same with the G229S mutant, which appeared to cause a complete loss-of-function after expression in mammalian cells (Figure 2), despite the seemingly innocuous location of Gly229 on the surface of the C-lobe of the kinase domain, where it was not obvious that amino acid replacement would affect any inter-domain interactions. Following purification on Ni^2+^-agarose, the WT and all mutant AMPK-α2 subunits co-purified with β2 and γ1 (Figure 5A), confirming that the mutations did not affect the formation of the α2β2γ1 complex. All mutants also appeared to be phosphorylated at Thr172 by LKB1, although the rates of phosphorylation of G229S, and in particular G167C, were much less than the others (Figure 5B) — note that the G167C mutant was not phosphorylated at Thr172 when expressed in mammalian cells either, although the G229S mutant was (Figure 4). When their activation by LKB1 was analysed, the S161F and G167C mutants were completely inactive, while the E168K mutant was activated normally. Although the G229S mutant was significantly activated at the highest concentration of LKB1, its AMPK activity was ∼80% lower than that of the wild type at all concentrations of LKB1 tested (Figure 5C).

We also examined the allosteric activation of naïve (unphosphorylated) human α2β2γ1 complexes by increasing concentrations of MK-8722, a pharmacological pan-β activator that binds at the ADaM site [48]. This reinforced the view that the G229S mutant showed a marked loss-of-fumction, while the S161F and G167C mutants were almost inactive, Interestingly, in this assay the E168K mutant also displayed a marked loss-of-function. Increasing concentrations of MK-8722 allosterically activated all mutants where the activity was measurable.

## Discussion

We will now present a brief analysis of the effect of selected mutations, focussing on those that appear to either reduce or increase AMPK function. Our analysis is based on the availability of structures for mammalian αβγ complexes [19,49] and on conservation of residues within the kinase domain compared with other protein kinases [50]:

1. G25D: this mutation caused a complete loss of kinase activity in HEK-293 cells (Figure 2A) and a failure to phosphorylate ACC1 in Hep-G2 cells (Figure 4). The mutation replaces the second glycine in the GXGXXGXV motif (sub-domain I [51]) of the kinase domain with a bulkier, negatively charged aspartate side chain. This motif is the ‘glycine-rich loop' in the small lobe of the kinase domain that caps the Mg.ATP binding site, with all three glycine residues (and the valine) being conserved in most protein kinases.
2. R63L: this mutation caused a reduced activation by phenformin in HEK-293 cells (Figure 2A), and is located in the C-helix, an α-helix within the small lobe of the α-KD whose conformation is critical in the activity of many kinases [52].
3. R72C: this mutation, which was reported in three different cases of skin cancer or melanoma, occurs in a loop of the kinase domain N-lobe, between the C-helix and the β4 strand. In two separate experiments, it caused a robust increase in activation by phenformin in HEK-293 cells. Interestingly, this arginine is conserved as R83 in human AMPK-α1, where it is one of three basic residues within or just after the C-helix that we previously proposed to interact with the phosphorylated ST loop of AMPK-α1, thus sterically hindering Thr172 phosphorylation by LKB1 [53]. The latter model would indeed predict that a non-conservative replacement of R72 might relieve inhibition of Th172 phosphorylation by the ST loop.
4. E100K: this mutant appeared to cause a complete loss of kinase activity in HEK-293 cells (Figure 2A) although it retained some ability to phosphorylate ACC1 in response to phenformin in Hep-G2 cells (Figure 4). This residue lies at the start of the D-helix in the kinase domain C-lobe, and interacts with the kinase inhibitor staurosporine in the structure of the α2β1γ1 complex [19]. The equivalent glutamate side chain in the catalytic subunit of cyclic AMP-dependent protein kinase (PKA-Cα) interacts with the ribose ring of Mg:ATP^2-^ bound in the catalytic site [54].
5. S161F: this mutation caused a total loss of kinase activity in HEK-293 cells (Figure 2A) and a greatly reduced ability to phosphorylate ACC1 in Hep-G2 cells (Figure 4). Ser161 occurs in the activation loop, just past the conserved DFG motif that defines the start of the loop. When expressed in Hep-G2 cells, the S161F mutant appeared to be maximally phosphorylated even in the absence of phenformin. When expressed in bacteria in the context of the recombinant α2β2γ1 complex, the mutant was phosphorylated apparently normally by LKB1 although this did not cause significant activation. This mutation therefore appears to disrupt the interactions between the phosphorylated activation loop and the remainder of the kinase domain, rather than inhibiting Thr172 phosphorylation *per se*.
6. G167C: this mutation, which lies close to the critical Thr172 phosphorylation site, caused a total loss of kinase activity in HEK-293 cells (Figure 2A) and a total loss of ACC1 phosphorylation in Hep-G2 cells (Figure 4). When expressed in Hep-G2 cells, Thr172 appeared not to be phosphorylated with this mutant, even in the presence of phenformin. When expressed in bacteria in the context of the recombinant α2β2γ1 complex, the mutant was phosphorylated more slowly than the wild type by LKB1, and this did not cause any significant activation. This mutation therefore appears to affect both phosphorylation by LKB1 as well as activation in response to Thr172 phosphorylation.
7. E168K: this mutation also occurs in the activation loop, just prior to the critical Thr172 phosphorylation site, and caused reduced activation by phenformin in HEK-293 cells in two independent experiments. In the context of a bacterially expressed α2β2γ1 complex, both Thr172 phosphorylation and activation by LKB1 appeared to be normal (Figure 5).
8. G229S: this mutation occurs at a surface location in the loop between the αG and αH helices of the kinase domain C-lobe. It appeared to cause a complete failure to be activated by phenformin when expressed in HEK-293 cells (Figure 2B), and for phenformin to increase phosphorylation of ACC1 in Hep-G2 cells, although phenformin increased Thr172 phosphorylation of this mutant relatively normally in Hep-G2 cells (Figure 4). This mutation is discussed further below.
9. R256Q: this kinase domain C-lobe mutation appeared to cause a complete loss of kinase activity in HEK-293 cells (Figure 2B) and a failure of phenformin treatment to phosphorylate ACC1 in Hep-G2 cells, although Thr172 phosphorylation was normal (Figure 4). An arginine is conserved in this position in most protein kinases (sub-domain XI [51]) and forms a conserved salt bridge with the glutamate residue of the APE motif at the C-terminal end of the activation loop (sub-domain VIII [51]). This interaction appears to anchor the activation loop to the remainder of the C-lobe.
10. D280N/S282F: these mutations, which appear to increase phenformin-stimulated kinase activity, lie at the N-terminal end of the AID that immediately follows the kinase domain and inhibits the latter in the absence of any other domains [45]. Why they cause increased activation is currently unclear.
11. S350F/M352I/D353N/P360S/H366Y/P367L/P367S/K399R: these mutations, which appear to cause a partial loss of activation by phenformin, are all in the α-linker, a sequence in extended conformation that binds to one surface of the regulatory adenine nucleotide-binding γ subunit. Indeed, P360, H366 and P367 are within the Regulatory Interaction Motif-2 (RIM2) that interacts directly with the activating nucleotide AMP when bound at the crucial CBS3 site [46]. It is therefore not surprising that mutations that interfere with the interactions made by the α-linker when AMP is bound might cause a partial loss of activation by the canonical (AMP-dependent) mechanism.
12. G405E: this mutation, which lies at the C-terminal end of the α-linker where it connects to the globular C-terminal domain (CTD), appeared to cause complete loss of kinase activity in HEK-293 cells (Figure 2D), although some residual ACC1 phosphorylation occurred in response to phenformin in Hep-G2 cells (Figure 4). The mutation also appeared to cause increased Thr172 phosphorylation in Hep-G2 cells even in the absence of phenformin.
13. E475K/E478K: these two ‘charge-swap' replacements, which substitute negatively charged for positively charged side chains, appear to increase activation in response to phenformin. The residues affected are located at the N-terminal end of the ST loop, a serine/threonine-rich loop that emerges from the α-CTD just prior to its final α-helix. This loop is either not resolved, or has been deliberately deleted, in constructs used to study crystal structures of the AMPK heterotrimer. The ST loop appears to be phosphorylated at multiple serine/threonine residues, which is reported to have negative effects on AMPK activation either due to inhibition of Thr172 phosphorylation [53,55] or to promotion of Thr172 dephosphorylation [56]. These phosphorylation sites lie close to Glu475 and Glu478, including Ser481 and Thr485 (Ser486 and Thr490 in α1) which are phosphorylated by GSK3 [56], and Ser491, which is subject to autophosphorylation in α2, while the equivalent residue in α1 (Ser496) is phosphorylated by Akt [53,55]. We have proposed that these phosphorylated residues interact with clusters of basic residues (including Lys62, Lys69, Arg72) that lie within or just following the αC-helix in the N-lobe of the kinase domain, and that this interaction may block the availability of Thr172 for phosphorylation by upstream kinases [53]. If Glu475 and Glu478 were also involved in interactions with the basic residues in the αC-helix that inhibited Thr172 phosphorylation, charge-swap mutations affecting these residues might well be expected to cause increased activation. Interestingly, the E480K mutation in AMPK-α1 that was also associated with an increased activation by phenformin (Figure 3C) is equivalent to the E475K mutation in AMPK-α2.

As pointed out in the introduction, there were particular ‘hot-spots' for mis-sense mutations in the *PRKAA2* gene, including the activation loop of the kinase domain, the AID, and the α-linker. In the cases of the activation loop and the α-linker, these were also hot-spots for loss-of-function effects, with seven out of nine mutations in the activation loop and 12 out of 18 mutations in the α-linker causing loss-of-function. However, this was not the case for the AID, where none of 11 mutations caused loss-of-function (Table 2).

With seven mis-sense mutations that appeared to cause total loss of kinase activity in HEK-293 cells, we also looked at their ability to phosphorylate the downstream target acetyl-CoA carboxylase (ACC1) in intact cells. For technical reasons explained earlier, this was performed in AMPK-α double knockout Hep-G2 cells rather than the double knockout HEK-293 cells used in Figures 2 and 3. Of the seven apparently kinase-inactive mutants tested, four (G25D, G167C, G229S and R256Q) failed to show any ACC1 phosphorylation even after treatment with phenformin. However, the other three (E100K, S161F and G405E) did appear to retain some ACC1 phosphorylation in response to phenformin, albeit at a much reduced level. Note that Ser80 on human ACC1 (Ser79 in rat ACC [57]) remains one of the most high affinity targets for AMPK [58], so that a large phosphorylation of ACC1 can occur in response to a very small increase in AMPK activity. Thus, ACC1 phosphorylation may be a much more sensitive assay to detect residual activity of mutants than direct kinase assays.

In the cases of three mutations within the activation loop of the kinase domain (close to Thr172) that appeared to cause either total (S161F/G167C) or partial (E168K) loss of phenformin activation in HEK-293 cells, plus one other (G229S) located in the C lobe that appeared to cause a total loss of phenformin activation, we also expressed them as α2β2γ1 complexes in *Escherichia coli* (when Thr172 is not phosphorylated) and incubated in cell-free assays with the purified LKB1 : STRADα : MO25α complex. Despite the fact that the S161F and G167C mutants were phosphorylated at Thr172 in cell-free assays by the LKB1 complex (albeit more slowly with G167C) there was little or no detectable activation of either mutant, indicating that with these mutants the phosphorylated activation loop fails to make the interactions required for activation. In contrast, the E168K mutant seemed to be phosphorylated and activated normally by LKB1 in cell-free assays. The results with the G229S mutant were interesting because although it was not activated by phenformin in HEK-293 cells it did appear to be phosphorylated at Thr172 and activated in the cell-free assays with the bacterially expressed α2β2γ1 complex, albeit much more slowly than the wild type. One interesting possibility is that Gly229 is involved in docking of LKB1 with AMPK so that the rate of Thr172 phosphorylation is reduced when it is mutated (although this was not evident using saturating concentrations of phenformin in Hep-G2 cells). Interestingly, a glycine residue is conserved at this position in AMPK-α1 and -α2 and in 11 out of 12 of the AMPK-related kinases that utilise LKB1 as upstream kinase [59,60], but not in many other protein kinases that utilise different upstream kinases. It would be of interest to mutate these glycine residues in some of the AMPK-related kinases and test their ability to be phosphorylated and activated by LKB1.

With the four bacterially expressed mutants studied in Figure 4, all that had measurable activity were allosterically activated by the ADaM site activator MK-8722, although their stimulated activities (including that of the E168K mutant) were still low compared with the wild type. This makes it perhaps unlikely that ADaM site activators would be efficacious in treatment of skin cancers carrying loss-of-function mutations in AMPK-α2.

In summary, of the 75 cases of human skin cancer or melanoma where mis-sense mutations in the *PRKAA2* gene were recorded and that we successfully expressed in HEK-293 cells (corresponding to 55 separate mutations, since some were listed multiple times), we found that 9 (12%) caused a total loss of AMPK activity, 18 (24%) caused a partial loss of phenformin-stimulated kinase activity, 11 (15%) caused an increase in phenformin-stimulated kinase activity, while 37 (49%) had no obvious effect on kinase activity. Adding in the fifteen nonsense, frameshift or splice site mutations (Table 1), which were all expected to cause a total loss-of function although this was not directly tested, there were 90 mutations in total with 24 (27%) either shown or expected to cause total loss-of-function, 18 (20%) causing a partial loss-of-function, 11 (12%) causing a gain-of-function and just 37 (41%) having no effect detectable in our assays. This is in marked contrast with the results for the *PRKAA1* gene encoding AMPK-α1, where just seven mis-sense mutations in total were reported in the cBioPortal database in skin cancer and melanoma in (Figure 1), with up to five of those having no apparent effect (Figure 3). This reinforces the idea that mutations causing loss-of-function in AMPK-α2 are being selected for in an isoform-specific manner in skin cancer and melanoma.

While this study was in progress, Zheng's group showed that knockout of *PRKAA2* in NF1-mutant melanoma cells promoted anchorage-independent growth in soft agar, while ectopic expression of *PRKAA2* in NF1- and AMPK-α2-deficient human melanoma cells greatly reduced this. In addition, knockout of *PRKAA2* in NF1-mutant melanoma cells increased their growth as xenografts in immunodeficient mice, and increased the presence of metastases in brain when they were administered by intracardiac injection [43]. These results strengthen the idea that AMPK-α2 is acting a tumour suppressor in the context of human melanoma, with loss-of-function mutations being selected for. However, that study did not directly address what proportion of the mutations that occur in melanoma and other types of skin cancer actually caused loss-of-function of AMPK-α2. Our results show that almost half (47%) of the mutations in the *PRKAA2* gene that have been reported in cases of human skin cancer and melanoma cause partial or total loss-of-function, reinforcing the hypothesis that such mutations are being selected for in these cancer types.

## Material and methods

### Antibodies and recombinant proteins

Phosphospecific antibodies against Thr172 on AMPK-α (Cat# 2535; RRID: AB_331250) and Ser79/212 on ACC1 (Cat# 11818; RRID: AB_2687505) were from Cell Signaling Technology. Total ACC1/ACC2 were detected using streptavidin conjugated to 800 nm fluorophore (Cat# S000-32, Rockland Immunochemicals). Anti-FLAG (Cat# F2426; RRID: AB_2616449) and anti-GAPDH (Cat# F9545; RRID: AB_796208) were from Sigma–Aldrich. Anti-AMPK-α (pan-α) antibody (Cat#ab32047; RRID: AB_722764) used for Western blotting was from AbCam.

DNAs encoding FLAG-tagged human AMPK-α1 or -α2 were generated as described previously [53].

A polycistronic plasmid expressing His_6_-tagged human AMPK (α2β2γ1 complex, a gift from AstraZeneca) was expressed in, and purified from, *Escherichia coli* as described previously [61]. Recombinant human LKB1:STRADα:MO25α complex was expressed in, and purified from, insect cells as described previously [62].

### Cell culture

AMPK-α1/-α2 double knockout HEK-293 cells were generated as described previously [63] and were grown in Dulbecco's Modified Eagle's Medium (25 mM glucose) containing 10% (v/v) foetal calf serum and 1% penicillin/streptomycin. They were grown in 6 cm dishes and when ∼40% confluent were transfected with DNAs encoding FLAG-tagged human AMPK-α1 or -α2 (wild type or mutant) using polyethylenimine (PEI) according to manufacturers’ instructions at a ratio of 3 : 1 (PEI : DNA). After 40 h, cells were transferred to medium with 5 mM glucose, and after a further 5 h were treated with or without phenformin for 1 h. They were then subject to rapid lysis *in situ* on the culture plate prior to kinase assays [64].

AMPK-α1/-α2 double knockout Hep-G2 cells were generated using the Cas9 D10A double nickase system. Pairs of guide RNAs targeted to exon 4 in both *PRKAA1* and *PRKAA2,* along with screening primers for genotyping, were designed and cloned by Thomas Macartney and are available by contacting MRCPPU Reagents and Services (https://mrcppureagents.dundee.ac.uk). The puromycin-selectable pBABED puro U6 vector was used for the sense guide RNA and the Cas9 D10A vector pX335 for the antisense guide RNAs. Hep-G2 cells were transfected with 1 µg of each of the four plasmids using Lipofectamine 3000 (ThermoFisher Scientific), according to the manufacturer's protocol. After transfection for 24 h, puromycin (2 µg/ml) was added in fresh medium, and again at 48 h. Selection was removed at 72 h and transfection was repeated without selection at 96 h to ensure high efficiency of knockout. After another 24 h, cells were single-cell sorted into individual wells of a 96-well plate coated with 0.1% gelatin in pre-conditioned Minimal Essential Medium (MEM) with 20% foetal bovine serum, 1× pyruvate and 1× non-essential amino acids. Clones were expanded and screened for loss of AMPK-α1 and -α2 protein by Western blotting. For the experiment shown in Figure 4, cells were grown in the same medium as HEK-293 cells. They were transfected for 48 h with plasmids encoding wild type or mutant FLAG-tagged AMPK-α2 subunits using Lipofectamine 3000 according to manufacturer's protocol. Cells were treated with or without phenformin for 1 h, lysed as for HEK-293 cells [64] and analysed by SDS–PAGE and Western blotting.

### Kinase assays

Cells were harvested by rapid detergent lysis on the culture plate [64], immunoprecipitated using anti-FLAG antibody and assayed using the *AMARA* peptide in resuspended immunoprecipitates as described previously [61], except that we used ^33^P- rather than ^32^P-labelled ATP.

### Site-directed mutagenesis

Mutations were created using the QuikChange Site-Directed Mutagenesis kit (Agilent). Mutations were confirmed by sequencing. DNA was then retransformed, amplified using midi- or maxi-preps, and reconfirmed by DNA sequencing.

### Phosphorylation and activation of bacterially expressed AMPK by LKB1

Bacterially expressed human α2β2γ1 AMPK complex or the indicated mutant (0.5 µg) was incubated for 15 min at 30°C with insect cell-expressed human LKB1:STRADα:MO25α complex (0, 2.23, 6.7 or 22.3 ng) with unlabelled ATP (200 µM) and MgCl_2_ (5 mM) in 50 mM Na Hepes buffer, pH 7.4, 200 mM NaCl, 1 mM dithiothreitol, 0.02% BRIJ-35 detergent, final volume of 25 µl. One aliquot (5 µl) was taken for AMPK kinase assay and the remaining 20 µl diluted with SDS–PAGE sample buffer, heated at 70°C for 10 min and 100 ng of protein analysed by SDS–PAGE.

### Other analytical procedures

Protein concentrations were estimated by Coomassie Blue binding [65]. SDS–PAGE was carried out using NuPAGE Bis-Tris or Tris-Acetate gels (ThermoFisher Scientific). Tris-Acetate gels were used for analysis of ACC and Bis-Tris gels for analysis of other proteins (AMPK and GAPDH).

### Statistical analysis

Statistical analysis was by two-way ANOVA using GraphPad Prism 9 for MacOS, with significance of differences in means being assessed using the Holm–Sidak multiple comparison test (*P < 0.05; **P < 0.01; ***P < 0.001; ****P < 0.0001). Error bars are ±SEM, with the number of replicates (dishes of cells) shown in Figure legends.

## Data Availability

All data relevant to this paper are included in the manuscript.

## Open Access

Open access for this article was enabled by the participation of University of Dundee in an all-inclusive *Read & Publish* agreement with Portland Press and the Biochemical Society under a transformative agreement with JISC.

## Abbreviations

ACC1: acetyl-CoA carboxylase-1/-α
ADaM: allosteric drug and metabolite
AID: auto-inhibitory domain
AMPK: AMP-activated protein kinase
CTD: C-terminal domain
KD: kinase domain
PEI: polyethylenimine
RIM2: regulatory interaction motif-2
