# Correction: Frequent loss-of-function mutations in the AMPK-α2 catalytic subunit suggest a tumour suppressor role in human skin cancers

**DOI:** 10.1042/BCJ20230380_COR

**Published:** 2025-12-05

It has come to the attention of the authors of the article “Frequent loss-of-function mutations in the AMPK-α2 catalytic subunit suggest a tumour suppressor role in human skin cancers” (DOI: 10.1042/BCJ20230380) that there is repetition of blots representing ACC1 in the upper and middle panels of Figure 4A. This was an unintentional error made during the assembly of the Figure. The raw data for Figure 4A have been identified, and it is confirmed that the incorrect data was used for the middle panel and a new Figure 4 with the correct data is provided here.

The raw data and requested correction have been assessed by and agreed with the Publisher. The authors apologise for the error and any inconvenience this may have caused.

**Figure 4:**
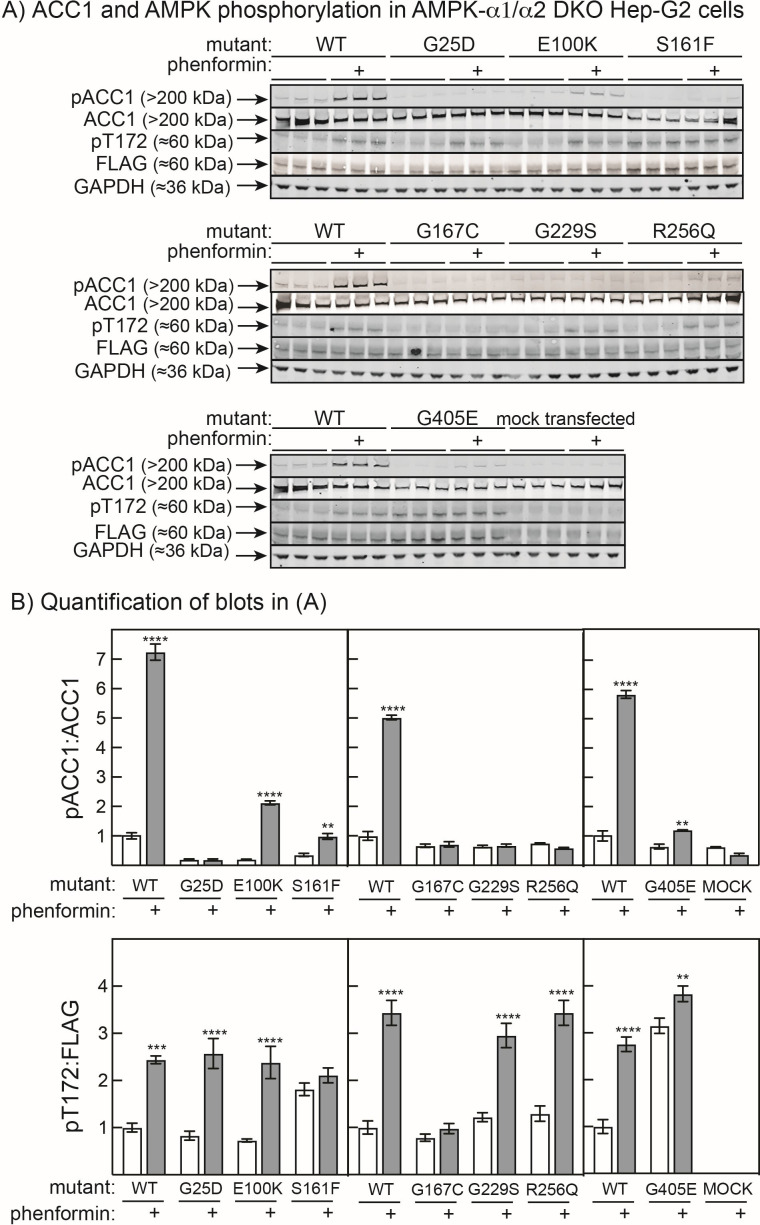
Phosphorylation of Ser80 on ACC and Thr172 in AMPK-α1/α2 double knockout Hep-G2 cells transfected with potentially inactive FLAG-tagged mutants.

